# Supplementary material for: Radiomic-based approaches in the multi-metastatic setting: a quantitative review
Source: BMC Cancer. 2025 Mar 25;25:538. doi: 10.1186/s12885-025-13850-5 (PMC11934564; doi:10.1186/s12885-025-13850-5)
Supplement: Supplementary file 1 — Supplementary Material 1. [file 12885_2025_13850_MOESM1_ESM.docx]

# Supplemental

## Literature Search (Specific Database Queries)

### Scopus

- TITLE-ABS ( radiomics AND ( metastas?s OR intertumor ) AND ( response OR survival ) ) AND ( LIMIT-TO ( SUBJAREA , "MEDI" ) ) AND ( LIMIT-TO ( DOCTYPE , "ar" ) ) AND ( LIMIT-TO ( LANGUAGE , "English" ) ) AND ( LIMIT-TO ( EXACTKEYWORD , "Radiomics" ) );

### PubMed

- (radiomics AND inter lesion heterogeneity);
- (radiomics AND metastases AND interlesion);
- (radiomics AND feature aggregation);

## Summary of Sample Sizes (Subgroup Analysis)

| **Subgroup** | **Dataset** | | |
| --- | --- | --- | --- |
|  | **TCIA - RADCURE** | **TCIA - CRLM** | **SARC021** |
| All patients | 3188 (2961) | 197 | 545 (325) |
| Patients with 2+ lesions | 2333 (3165) | 127 | 407 (288) |
| Patients with 3+ lesions | 2016 (1883) | 64 | 151 (121) |

**Table S1:** *Summary of sample sizes for various subgroup analyses. For the TCIA - RADCURE and SARC021 datasets, numbers in parentheses represent the number of patients with a primary tumor and with a lung metastasis, respectively.*

## Numerical Results

|  | **RADCURE (n=3188)** | | **TCIA-CRLM (n=197)** | | **SARC021 (n=545)** | |
| --- | --- | --- | --- | --- | --- | --- |
|  | **Train (Median (95% CI))**  **(n=2550)** | **Test**  **(n=638)** | **Train (Median (95% CI)) (n=157)** | **Test (n=40)** | **Train (Median (95% CI))**  **(n=436)** | **Test**  **(n=109)** |
| **Largest Lesion** | 0.599 (0.570-0.634) | 0.593 | 0.664 (0.601-0.739) | 0.658 | 0.622 (0.579-0.659) | 0.619 |
| **Largest Lesion**  **+ Number of Mets** | 0.595 (0.549-0.630) | 0.595 | 0.653 (0.583-0.733) | 0.622 | 0.621 (0.577-0.659) | 0.675 |
| **Smallest Lesion** | 0.556 (0.530-0.585) | 0.545 | 0.656 (0.575-0.732) | 0.627 | 0.593 (0.544-0.633) | 0.587 |
| **Primary Tumor*** | 0.656 (0.625-0.683) | 0.611 | - | - | - | - |
| **Largest Lung Lesion*** | - | - | - | - | 0.576 (0.523-0.632) | 0.627 |
| **Weighted Average (N-largest)** | 0.599 (0.570-0.634) | 0.593 | 0.664 (0.601-0.739) | 0.658 | 0.622 (0.579-0.659) | 0.619 |
| **Concatenation** | 0.599 (0.570-0.634) | 0.593 | 0.664 (0.601-0.739) | 0.658 | 0.622 (0.579-0.659) | 0.619 |
| **Cosine Metrics** | N/A | | | | | |
| **Unweighted Average** | 0.600 (0.572-0.645) | 0.632 | 0.675 (0.577-0.752) | 0.687 | 0.605 (0.567-0.653) | 0.648 |
| **Weighted Average** | 0.607 (0.577-0.640) | 0.600 | 0.665 (0.594-0.737) | 0.644 | 0.618 (0.576-0.662) | 0.682 |

**Table S2:** *Comparison of feature aggregation methods for all patients, irrespective of number of tumors, in three distinct metastatic settings. The Cox proportional hazards model was used to fit the data. The training set was bootstrapped to generate confidence intervals (CI) for model performance; the metric used for model performance is the Concordance Index (C-Index).*

|  | **RADCURE (n=2333)** | | **TCIA-CRLM (n=127)** | | **SARC021 (n=407)** | |
| --- | --- | --- | --- | --- | --- | --- |
|  | **Train (Median (95% CI))**  **(n=1868)** | **Test**  **(n=465)** | **Train (Median (95% CI)) (n=102)** | **Test (n=25)** | **Train (Median (95% CI))**  **(n=323)** | **Test**  **(n=84)** |
| **Largest Lesion** | 0.614 (0.584-0.649) | 0.604 | 0.702 (0.623-0.785) | 0.681 | 0.611 (0.571-0.649) | 0.633 |
| **Largest Lesion**  **+ Number of Mets** | 0.597 (0.572-0.630) | 0.587 | 0.669 (0.583-0.747) | 0.609 | 0.617 (0.573-0.649) | 0.677 |
| **Smallest Lesion** | 0.597 (0.563-0.628) | 0.551 | 0.658 (0.578-0.741) | 0.702 | 0.618 (0.576-0.674) | 0.623 |
| **Primary Tumor*** | 0.570 (0.536-0.605) | 0.595 | - | - | - | - |
| **Largest Lung Lesion*** | - | - | - | - | 0.605 (0.539-0.667) | 0.602 |
| **Weighted Average (N-largest)** | 0.613 (0.587-0.642) | 0.615 | 0.727 (0.638-0.826) | 0.732 | 0.603 (0.556-0.659) | 0.625 |
| **Concatenation** | 0.579 (0.543-0.612) | 0.576 | 0.699 (0.613-0.797) | 0.689 | 0.600 (0.550-0.658) | 0.614 |
| **Cosine Metrics** | 0.500 (0.500-0.500) | 0.500 | 0.500 (0.500-0.531) | 0.500 | 0.500 (0.500-0.500) | 0.500 |
| **Unweighted Average** | 0.607 (0.578-0.643) | 0.614 | 0.745 (0.656-0.858) | 0.689 | 0.630 (0.583-0.679) | 0.671 |
| **Weighted Average** | 0.650 (0.619-0.670) | 0.623 | 0.740 (0.653-0.842) | 0.821 | 0.609 (0.556-0.656) | 0.646 |

**Table S3:** *Comparison of feature aggregation methods for patients with two or more tumors, in three distinct metastatic settings. The Cox proportional hazards model was used to fit the data. The training set was bootstrapped to generate confidence intervals (CI) for model performance; the metric used for model performance is the Concordance Index (C-Index).*

|  | **RADCURE (n=2016)** | | **TCIA-CRLM (n=64)** | | **SARC021 (n=151)** | |
| --- | --- | --- | --- | --- | --- | --- |
|  | **Train (Median (95% CI))**  **(n=1613)** | **Test**  **(n=403)** | **Train (Median (95% CI)) (n=51)** | **Test (n=13)** | **Train (Median (95% CI))**  **(n=121)** | **Test**  **(n=30)** |
| **Largest Lesion** | 0.587 (0.558-0.617) | 0.589 | 0.779 (0.659-0.886) | 0.821 | 0.615 (0.532-0.690) | 0.627 |
| **Largest Lesion**  **+ Number of Mets** | 0.587 (0.557-0.617) | 0.582 | 0.797 (0.674-0.912) | 0.804 | 0.615 (0.525-0.690) | 0.672 |
| **Smallest Lesion** | 0.578 (0.544-0.609) | 0.531 | 0.744 (0.625-0.849) | 0.857 | 0.630 (0.549-0.719) | 0.748 |
| **Primary Tumor*** | 0.594 (0.566-0.626) | 0.590 | - | - | - | - |
| **Largest Lung Lesion*** | - | - | - | - | 0.622 (0.550-0.704) | 0.572 |
| **Weighted Average (N-largest)** | 0.606 (0.568-0.637) | 0.596 | 0.793 (0.653-0.909) | 0.768 | 0.636 (0.570-0.693) | 0.588 |
| **Concatenation** | 0.568 (0.530-0.603) | 0.617 | 0.756 (0.553-0.864) | 0.821 | 0.673 (0.586-0.739) | 0.598 |
| **Cosine Metrics** | 0.520 (0.496-0.555) | 0.516 | 0.595 (0.456-0.756) | 0.554 | 0.532 (0.473-0.605) | 0.625 |
| **Unweighted Average** | 0.609 (0.574-0.645) | 0.627 | 0.818 (0.670-0.939) | 0.839 | 0.618 (0.544-0.701) | 0.674 |
| **Weighted Average** | 0.615 (0.581-0.648) | 0.586 | 0.842 (0.714-0.982) | 0.982 | 0.615 (0.545-0.681) | 0.674 |

**Table S4:** *Comparison of feature aggregation methods for patients with three or more tumors, in three distinct metastatic settings. The Cox proportional hazards model was used to fit the data. The training set was bootstrapped to generate confidence intervals (CI) for model performance; the metric used for model performance is the Concordance Index (C-Index).*
